# Conservative Surgery for Early Breast Cancer

**Published:** 1989-05

**Authors:** R. E. May, D. A. Gillatt, Sally Goodman, Elizabeth Whipp, R. Laing

**Affiliations:** Consultant Surgeon Department of General Surgery and the Bristol Radiotherapy Centre, Frenchay Hospital Correspondence to: Mr. R. E. May Department of Surgery, Frenchay Hospital; Registrar in Surgery Department of General Surgery and the Bristol Radiotherapy Centre, Frenchay Hospital Correspondence to: Mr. R. E. May Department of Surgery, Frenchay Hospital; Senior Registrar in Radiotherapy Department of General Surgery and the Bristol Radiotherapy Centre, Frenchay Hospital Correspondence to: Mr. R. E. May Department of Surgery, Frenchay Hospital; Consultant Radiotherapist Department of General Surgery and the Bristol Radiotherapy Centre, Frenchay Hospital Correspondence to: Mr. R. E. May Department of Surgery, Frenchay Hospital; Senior House Officer in Surgery Department of General Surgery and the Bristol Radiotherapy Centre, Frenchay Hospital Correspondence to: Mr. R. E. May Department of Surgery, Frenchay Hospital

## Abstract

Wide local excision followed by radiotherapy was offered as an alternative to mastectomy to 93 women on 96 occasions. Complications attributable to the surgery occurred in 4 per cent whilst 37.5 per cent developed complications secondary to the radiotherapy. The complications were generally of a mild nature. Nine patients (9.3%) have developed regional recurrence and seventeen (17.7%) distant metastases with a mean follow up of 53.4 months.


### Conservative Surgery for Early Breast Cancer Results from a District Hospital

**R. E. May MS, FRCS**
Consultant Surgeon

**D. A. Gillatt FRCS**
Registrar in Surgery

**Sally Goodman MRCP, FRCR**
Senior Registrar in Radiotherapy

**Elizabeth Whipp FRCR**
Consultant Radiotherapist

**R. Laing MB, ChB**
Senior House Officer in Surgery

Department of General Surgery and the Bristol Radiotherapy Centre, Frenchay Hospital
Correspondence to: Mr. R. E. May Department of Surgery, Frenchay Hospital.

### SUMMARY
Wide local excision followed by radiotherapy was offered as an alternative to mastectomy to 93 women on 96 occasions. Complications attributable to the surgery occurred in 4 per cent whilst 37.5 per cent developed complications secondary to the radiotherapy. The complications were generally of a mild nature. Nine patients (9.3%) have developed regional recurrence and seventeen (17.7%) distant metastases with a mean follow up of 53.4 months.

### INTRODUCTION
As recently as 1985 a survey of the treatment of primary breast cancer in the United Kingdom showed that 84% of surgeons still preferred mastectomy [1]. Many surgeons are reluctant to adopt partial mastectomy or local excision because they fear an increased incidence of local recurrence. There has been however, over a number of years, increasing interest in conserving the breasts of women with early disease.

The pioneer work of Keynes was set out in a number of papers between 1927 and 1937 [2, 3]. He treated both primary and recurrent tumours by conservative surgery and radium needle implants, with a local recurrence rate of 8%. Atkins [4] reported the results of a trial of wide excision (extended tylectomy) and radiotherapy; Crile [5] in the United States advocated partial mastectomy rather than radical surgery; and in France Calle [6] and Pierquin [7], were treating patients with excision biopsy and radiotherapy after the pioneering work of Baclesse [8] in the late 1940s and early 1950s.

In the mid-seventies some patients in our unit with medially placed lesions underwent local excision followed by radiotherapy. In the late 1970s one author (REM), following discussion with radiotherapy colleagues, decided to recommend treatment with local excision and radiotherapy to patients with early breast cancer. In particular for peripherally situated tumours which appeared to be 5 cms or less in diameter.

We report the results of conservative treatment in the first 93 women treated in this way for 96 carcinomas, and followed up for a median period of 53.4 months. We have attempted to assess the morbidity of treatment in detail as well as the rate of local recurrence and development of distant metastases.

### PATIENTS AND METHODS
The case notes of 93 women with clinical stage 1 or early stage 2 breast cancer were reviewed. These women had tumours measuring two centimetres diameter or less on clinical examination and mammography and the diagnosis of carcinoma was made by fine needle aspiration biopsy. Patients were counselled by the surgeon in the outpatient clinic and offered local excision as an alternative to mastectomy. Three women subsequently developed a second primary tumour in the opposite breast and were treated in the same way, making a total of 96 primary tumours. Over the six year period an increasing proportion of patients were treated with lumpectomy [1].

Surgery consisted of local excision of the tumour along with a 2 cm margin of normal tissue. The skin incision was placed to give the best possible cosmetic result. The wound was closed with a non-absorbable skin suture and drained for 24 hours with a suction drain. Axillary sampling was not undertaken routinely. On histological confirmation of the cytological diagnosis the patient was referred to the radiotherapy clinic.

Most patients were treated by two consultants who used similar radiotherapy techniques. Just under 20% were treated by other consultants or with different techniques, particularly in the early part of the period under review. Over 80% were treated on mega-voltage x-ray equipment with tangential opposed fields encompassing the breast and cervico-axillary chain. The scar and tumour bed received a boost dose. The large volume was treated daily over 4 to 5 weeks, the boost site over to 2 to 5 days.

To enable comparison of cases, TDF (tumour/dose/fractionation) factors have been calculated according to the formula of Orton and Ellis [9] and table 1 shows the minimum TDF factor in the tumour volume and the maximum TDF factor in the boost volume. In practice the most commonly used phase of fractionation was 4,600 cGy in 20 fractions over 4 weeks followed by a boost of 1,200 cGy in 5 fractions in one week. Over the past 5 years chest wall thickness has been measured using CT or US scanning. This has enabled the dose given to the underlying lung to be reduced.

**Table 1**

**Radiation doses**

| Numbers | *Minimum TDF in treatment volume* | Numbers | *Maximum TDF in Tumour bed* |
|---|---|---|---|
| 51 | 82 | 21 | 101–110 |
| 24 | 93 | 21 | 111–120 |
| 9 | 100 | 7 | 121–130 |
| 4 | >100 | 5 | >130 |
| 8 | not calculable* | 42 | not calculable but in excess of 93* |

* Treatment method makes accurate calculation impossible.

### RESULTS

Ninety three women aged between 28 and 80 years (mean 46.1 years) were treated by wide local excision of tumour followed by radiotherapy (table 2). Three women had already undergone mastectomy, and three subsequently underwent conservative surgery for primary tumour affecting the opposite breast. Two patients have been lost to follow up (6 and 48 months). The mean follow-up on the remaining patients is 53.4 months (range 9 months—136 months).

**Table 2**

**Details of 93 treated patients**

*Age mean 46.1 years (range 28–80 years)*

| | |
|---|---|
| Unilateral tumour | 90 |
| Bilateral tumour | 6* |
| Mean follow up 53.4 months (range 9–136 months) | |

* Three patients had a previous mastectomy, three had bilateral tumours treated by local excision and radiotherapy.

After histological examination the tumours were restaged as follows: 50 as pT1, 43 as pT2, 3 as pT3 (table 3). Lymph nodes containing tumour were found in the resection specimens from five patients with tumours of the upper outer quadrant.

**Table 3**

**Pathology: 96 tumours in 93 women**

| | | |
|---|---|---|
| Stage of lesion | pT1 | 50 (52%) |
| | pT2 | 43 (45%) |
| | pT3 | 3 (3%) |
| Histology | Invasive ductal | 88 |
| | Lobular | 3 |
| | Medullary | 2 |
| | Mucoid | 2 |
| | Clear cell | 1 |
| | | 96 |
| Axillary lymph nodes containing tumour | | 5 |

Most of the tumours were carcinomas of ductal origin. There were small numbers of lobular, medullary, mucoid and clear cell carcinomas (table 3).

Four patients developed acute complications directly attributable to the surgical procedure (table 4). Two developed haematoma despite drainage and there was one case of partial wound dehiscence after early suture removal. One patient developed a post-operative pneumonia requiring antibiotic treatment and physiotherapy.

Radiotherapy produced two groups of complications: early and late. Because the study was retrospective these could not be assessed in detail. In particular, the relative contributions of surgery and radiotherapy to chronic changes was difficult to assess.

**Table 4**

**Complications in 93 patients receiving treatment for 96 tumours**

| | | |
|---|---|---|
| *Acute* | | |
| Surgery | Wound haematoma | 2 |
| | Wound dehiscence | 1 |
| | Chest infection | 1 |
| | | 4 |
| Radiotherapy | Frozen shoulder | 6 |
| | Breast oedema | 9 |
| | Radiation pneumonitis | 4 |
| | | 19 |
| Chronic | Lymphoedema | 5 |
| | Chest wall pain | 12 |
| | Pulmonary fibrosis (limited) | 3 |
| | Breast fibrosis | 23 |
| | | 43 (36 patients) |

Most patients developed an acute skin reaction which in a very small number reached the stage of moist desquamation. The most severe example occurred with unconventional treatment and a high TDF factor. Acute radiation pneumonitis producing mild symptoms occurred in 4 patients. These reactions settled completely with conservative measures.

More problems were caused by stiff and painful shoulders, chest wall pain, and oedematous painful breasts. In most patients symptoms improved with physiotherapy, reassurance, analgesia and time, but in a small number they became chronic. Persistence of symptoms was usually, but not always, related to the degree of induration in the breast and around the axilla. Mild lymphoedema of the arm developed in 5 cases, 2 of whom had undergone axillary dissection. Limited pulmonary fibrosis was seen on chest x-ray in 3 women but did not cause symptoms.

Follow-up assessment for recurrent disease could be difficult if there was oedema or fibrosis affecting the whole breast or the scar area. Doubt about possible recurrence was expressed in 31 patients on at least one occasion.

One patient developed neuralgic amyotrophy, and another a pericardial effusion. These were related temporally, but not necessarily causally, to their treatment. Both conditions resolved completely. Three patients developed depression about their illness or their symptoms sufficient to warrant treatment.

Most patients had a good or acceptable cosmetic result. A small number with a central tumour or small breasts had distortion of the nipple but only one patient requested surgical correction.

Nine patients have developed local recurrence so far: 7 in the breast, 2 in the axilla. Three were confirmed histologically: one after mastectomy, the others after local excision. One woman had 2 local recurrences excised, at 18 months and 4 years, and remains well 7 years later. Five patients with clinical evidence of local recurrence were treated with hormones and one with further radiotherapy. There were 3

complete responses and one partial response. Local response was not recorded in two patients who developed synchronous metastatic disease.

Seventeen patients have developed distant metastases in multiple sites: 10 in bone, 4 in liver, 6 in lung, 2 in skin, and 6 in other sites.

Nine patients have died, 8 as a result of their disease and one of unrelated cardiovascular disease. Three women with local recurrence died of metastatic disease, which in 2 cases developed synchronously. The remaining 6 women with local recurrence remain free of metastatic disease at the time of review (currently 7 to 84 months, mean 44 months).

### DISCUSSION

The aims of treatment for breast cancer are twofold: to prevent local recurrence and to reduce the chance of development of metastatic disease. It seems that the method of treating the primary tumour, as long as local control is achieved, has no influence on whether or not metastases develop [10].

However the best way to achieve local control has produced fierce debate for nearly a century. The radical approaches of Halsted and Patey have been replaced by simple mastectomy, lymph node sampling and radiotherapy if appropriate. An increasing number of clinicians now see mastectomy itself as unnecessary for local control of disease—an approach pioneered 50 years ago by Keynes.

There are many different approaches to local treatment of breast cancer. These include local excision, wide local excision, lumpectomy, quadrantectomy, partial mastectomy and tylectomy. This proliferation of undefined terms is confusing and makes comparison of results from different centres difficult. We have used the terms adopted at a recent international workshop [11]. Thus *wide local excision* is removal of the tumour with a margin of normal tissue. More extensive excision is classified as quadrantectomy and cases treated by this technique are not included in this series.

Local excision of tumour alone is followed by local recurrence in an unacceptably high number of cases [12]. A high proportion of breast cancers are found to be multicentric on pathological examination of mastectomy specimens [13, 14, 15]. This has strengthened the argument for mastectomy. However, recent reports have shown that a low rate of local recurrence, comparable with that achieved by radical mastectomy, can be achieved by a combination of local excision and radiotherapy [16, 17, 18]. This approach removes the primary tumour and treats other areas in the breast, which may have malignant potential. These include the scar, which may be contaminated with viable tumour cells. It is important that macroscopic tumour is completely excised. The dose of radiotherapy must be adequate and applied to the whole breast [19]. Inadequate irradiation almost certainly explains the poor results of conservative treatment in a large trial [4].

Local recurrence after conservative treatment does not appear to carry a poor prognosis [10]. Radical treatment of locally recurrent disease is usually effective. One case developed local recurrence at 18 months and again at 4 years, treated each time by local excision. She remains well without metastatic disease at 11 years from diagnosis.

There is no evidence that conservative treatment adversely affects prognosis. However, most authors have compared their results with historical controls or with those from other centres. The 5 year results of a major randomised study of mastectomy versus lumpectomy with radiotherapy published recently by the National Surgical Adjuvant Breast Project (1987) support the premise thast conservative surgery does not worsen prognosis. The Cancer Research Campaign launched a prospective randomised trial in 1982. Unfortunately this trial was slow to recruit patients and closed prematurely. The difficulties of obtaining fully informed consent were largely responsible for the early closure (letter to participants).

With any treatment for cancer, the quality of life achieved is important. Mastectomy has a high complication rate: in one study, 17% of time spent in hospital was because of complications [21]. One third of cases developed seromas, and other problems included painful scars, chest wall pain and lymphoedema. Side effects are more severe and more common after more radical forms of mastectomy, or if post-operative radiotherapy is given. In our series the majority of the problems were minor or transient, and were, in the early stages at least, largely due to the radiotherapy.

There has been much interest in the psychological problems of patients with breast cancer. In one series a quarter of the women developed clinical depresion within one year of mastectomy [22]. Conservative treatment is widely thought to lessen the psychological morbidity but a recent report found no difference between women undergoing mastectomy and those having conservative treatment [23]. The question of patient satisfaction, as opposed to psychological morbidity, is harder to assess formally, and there is little information available. In general, women express satisfaction with conservative treatment even when the cosmetic results are less than perfect, and our patients were no exception. The precise psychological impact of the various forms of treatment requires further formal study.

In most of our patients the diagnosis was made by fine needle aspiration biopsy. This enabled discussion of the diagnosis, and possible approaches to treatment, in the outpatient clinic. There were no false positive results, and no frozen sections were required during surgery. This, and the shorter time required for local excision, could ease pressure on the surgical services. The inpatient stay was reduced from more than 7 days for mastectomy cases to around 48 hours after local excision. The corollary of this is more demand on radiotherapy planning and treatment time and there may be little overall cost benefit to the NHS. The side effects of radical radiotherapy to the whole breast were numerous but usually minor. With increasing experience of treating the intact breast, and with attention to treatment planning and dose, the more serious late side effects, which may produce permanent morbidity are rarely seen.

In the absence of any clear difference in survival, morbidity or cost, there is no need for a mutilating operation. In the future it may be possible to select patients for whom radiotherapy is not necessary, and then the true cost benefit may be seen, together with a significant reduction in morbidity.

### CONCLUSION

We report the results of 96 cases of early breast cancer treated by a wide local excision and radical radiotherapy. These are preliminary results and long term follow-up will be necessary before we can draw any conclusions about an effect on survival. At present we find a local recurrence rate of 9% encouraging and will continue to offer this treatment option to women with early breast cancer.

### REFERENCES

1. GAZET, J. C., RAINSBURY, R. M., FORD, H. T., POWLES, T. J. and COOMBES, R. C. (1985) Survey of treatment of primary breast cancer in Great Britain. *Br.Med.J.* **290**, 1793–5.
2. KEYNES, G. (1927) The radium treatment of carcinoma of the breast. *St.Barts. Hosp.Rep.* **60**, 91–95.
3. KEYNES, G. (1937) Conservative treatment of cancer of the breast. *Br.Med.J.* **2**, 643–647.
4. ATKINS, H., HAYWARD, J. L., KLUGMAN, D. J., WAYNE, A. B. (1972) Treatment of Early Breast Cancer: A report after ten years of a clinical trial. *Br.Med.J.* **2**, 423–429.
5. CRILE, G., ESSELSTYN, C. B., HERMANN, R. E., HOERR, S. O. (1973) Partial mastectomy for carcinoma of the breast. *Surg. Gynaecol. Obstet.* **136**, 929–933.

6. CALLE, R., PILLERON, J. P., SCHLIENGER, P. and VILCOQ, J. B.(1978) Conservative management of operable breast cancer. Ten years experience at the Foundation Curie. *Cancer* **42**, 2045.
7. PIERQUIN, B., MUELLER, W., BAILLET, F. *et al* (1978) Radical radiation therapy for cancer of the breast, the experience of Creteil. Frontiers of Radiation therapy and Oncology **12**, 150.
8. BACLESSE, F., ENNUYER, A. and CHEGUILLAUME, J. (1960) Est-on authorise a pratique une tumourectomie simple suivie de radiotherapie en case de tumuer mammaire? *Journal de Radiologie et Electrologie* **41**, 137.
9. ORTON, C. G. and ELLIS, F. (1973) A simplification in the use of the NSD concept in practical radiotherapy. *Br. J. Radiol.* **46**, 529–537.
10. PIERQUIN, B., BAILLET, F. and WILSON, J. F. (1976) Radiation therapy in the management of primary breast cancer. *Am. J. Roentgenol.* **127**, 645.
11. HARRIS, J. R., HELLMAN, S., and KINNE, D. W. (1985) Limited surgery and radiotherapy for early breast cancer. *N.Engl.J.Med.* **313**, 1365–8.
12. TAGART, R., BRATHERTON, D., HARTLEY, L. *et al* (1985) Partial mastectomy alone in early breast cancer. *Br.Med.J.* **290**, 434.
13. CAHILL, C. J., GIBBS, N. M., BOULTER *et al* (1983) Invasive breast cancer—the tip of the ice-berg. *Ann.Roy.Coll.Surg.* **65**, 356–359.
14. TELLEM, M., PRIVE, L., and MERANZE, D. R. (1962) Four quadrant study of breasts removed for carcinoma. *Cancer* **15**, 10–17.
15. QUALHEIM, R. E. and GALL, E. A. (1957) Breast carcinoma with multiple sites of origin. *Cancer* **10**, 460–468.
16. VERONESI, U., SACCOZZI, R., DEL VECCHIO, M. *et al* (1981) Comparing radical mastectomy with quadrantectomy, axillary dissection and radiotherapy in patients with small cancers of the breast. *N.Engl.J.Med.* **305**, 6–11.
17. FISHER, B., BAUER, M., MARGOLESE, R. *et al* (1985) Five year results of a randomised clinical trial comparing total mastectomy and segmental mastectomy with or without radiation in the treatment of breast cancer. *N.Engl.J.Med.* **312**, 665–673.
18. BULMAN, A. S., ZEITMAN, A., PHILLIPS, R. H. *et al* (1987) Interim results of treatment of breast cancer with breast conservation for all patients. *Surgery* **101**, 395–9.
19. FLETCHER, G. H. (1972) Local results of irradiation in the primary management of localised breast cancer. *Cancer* **29**, 545–551.
20. FENTIMAN, I. S., MATTHEWS, P. N., DAVISON, O. W. (1985) Survival following local skin recurrence after mastectomy. *Br.J.Surg.* **72**, 14–16.
21. TEJLER, G. and ASPEGREN, K. (1985) Complications and hospital stay after surgery for breast cancer: a prospective study of 385 patients. *Br.J.Surg.* **72**, 542–544.
22. MAQUIRE, G. P., LEE, E. G., BEVINGTON, D. J. (1978) Psychiatric problems in the first year after mastectomy. *Br.Med.J.* **i**, 963–965.
23. FALLOWFIELD, L. J., BAUM, M., and MAQUIRE, G. P. (1986) Effects of breast conservation on psychological morbidity associated with diagnosis and treatment of early breast cancer. *Br.Med.J.* **293**, 1331–1334.

**ACKNOWLEDGEMENT**

We should like to thank Mr. Noel Pizey FFR FRCS whose enthusiasm encouraged evaluation of this treatment. We would also like to thank Mr. L. R. Celestin for allowing us to include some of his patients.

